# Supplementary material for: A Critical View of the Application of the APEX Software (Aqueous Photochemistry of Environmentally-Occurring Xenobiotics) to Predict Photoreaction Kinetics in Surface Freshwaters
Source: Molecules. 2019 Dec 18;25(1):9. doi: 10.3390/molecules25010009 (PMC7017383; doi:10.3390/molecules25010009)
Supplement: Supplementary file 1 [file molecules-25-00009-s001.zip › 3.2.4_gcc-4.4.0.zip!/3.2.4_gcc-4.4.0/3.2.4_gcc-4.4.0/doc/octave/html/interpreter/Three_002ddimensional-Geometric-Shapes.html]

Three-dimensional Geometric Shapes - Untitled


Previous: Three-dimensional Function Plotting,
Up: Three-Dimensional Plotting


---

##### 15.1.2.2 Three-dimensional Geometric Shapes

— Function File: **cylinder**  
— Function File: **cylinder** (r)  
— Function File: **cylinder** (r, n)  
— Function File: [x, y, z] = **cylinder** (...)  
— Function File: **cylinder** (ax, ...)  
> Generates three matrices in `meshgrid` format, such that
> `surf (`x`,` y`,` z`)` generates a unit cylinder.
> The matrices are of size n`+1`-by-n`+1`.
> r is a vector containing the radius along the z-axis.
> If n or r are omitted then default values of 20 or [1 1]
> are assumed.
>
> Called with no return arguments, `cylinder` calls directly
> `surf (`x`,` y`,` z`)`. If an axes handle ax
> is passed as the first argument, the surface is plotted to this set
> of axes.
>
> Examples:
>
> ```
>           disp ("plotting a cone")
>           [x, y, z] = cylinder (10:-1:0,50);
>           surf (x, y, z);
> ```
>
>
>
>
> **See also:** sphere.

— Function File: [x, y, z] = **sphere** (n)  
— Function File: **sphere** (h, ...)  
> Generates three matrices in `meshgrid` format, such that
> `surf (`x`,` y`,` z`)` generates a unit sphere.
> The matrices of n`+1`-by-n`+1`. If n is
> omitted then a default value of 20 is assumed.
>
> Called with no return arguments, `sphere` call directly
> `surf (`x`,` y`,` z`)`. If an axes handle is passed
> as the first argument, the surface is plotted to this set of axes.
>
>
>
> **See also:** peaks.

— Function File: [x, y, z] = **ellipsoid** (xc,yc, zc, xr, yr, zr, n)  
— Function File: **ellipsoid** (h, ...)  
> Generate three matrices in `meshgrid` format that define an
> ellipsoid. Called with no return arguments, `ellipsoid` calls
> directly `surf (`x`,` y`,` z`)`. If an axes handle
> is passed as the first argument, the surface is plotted to this
> set of axes.
>
>
>
> **See also:** sphere.
